# Insecticide resistance level and geographical distribution of target-site mutations in field collections of the house fly (*Musca domestica*) from Türkiye

**DOI:** 10.1007/s10646-026-03107-7

**Published:** 2026-06-18

**Authors:** Nafiye Koç-İnak, Neslihan Akkaya, Eneshan Sarıkaya, Esra Ünlüce, Duran Yakup Türkmen, Sevimnur Durmaz, Ömer Akkaya, Levent Altıntaş, Emre İnak

**Affiliations:** 1 Department of Parasitology, Faculty of Veterinary Medicine, Ankara University, Diskapi 06070 Ankara, Türkiye; 2 Department of Pharmacology and Toxicology, Graduate School of Health Sciences, Ankara University, Diskapi 06070 Ankara, Türkiye; 3 Department of Parasitology, Graduate School of Health Sciences, Ankara University, Diskapi 06070 Ankara, Türkiye; 4 Department of Food Hygiene and Technology, Graduate School of Health Sciences, Afyon Kocatepe University, 03200 Afyonkarahisar, Türkiye; 5 Department of Pharmacology and Toxicology, Faculty of Veterinary Medicine, Ankara University, Diskapi 06070 Ankara, Türkiye; 6 Department of Plant Protection, Faculty of Agriculture, Ankara University, Diskapi 06110 Ankara, Türkiye

**Keywords:** *Musca domestica*, Insecticide resistance, Target-site mutations, Molecular docking, Acetylcholinesterase

## Abstract

The house fly, *Musca domestica* (Linnaeus, 1758), is a major threat to public health and food safety due to its ability to transmit numerous pathogenic microorganisms. Despite global control efforts, populations remain abundant in residential areas, livestock facilities, and slaughterhouses, largely due to their high reproductive potential and widespread insecticide resistance. Continuous monitoring of resistance and its underlying mechanisms is therefore essential. In this study, toxicity bioassays were conducted on five strains collected from slaughterhouses using the feeding method. While permethrin, deltamethrin, and bendiocarb were ineffective, thiamethoxam showed variable susceptibility, and fipronil exhibited the highest efficacy. In addition, target-site mutations associated with resistance were screened in a total of 35 samples collected from slaughterhouses and residential areas. A high prevalence of T929I and L1014F mutations in the voltage-gated sodium channel (VGSC) and V260L, A316S, G342A/V, and F407Y mutations in acetylcholinesterase (AChE) — the target sites of pyrethroids and carbamates, respectively — was detected. In silico docking of bendiocarb on *M. domestica* AChE was conducted for the first time and largely confirmed the functional impact of *ace* mutations through reduced binding energies. Notably, the L1014H (VGSC) and G342V (AChE) mutations occurred exclusively in slaughterhouse samples, potentially indicating stronger local selection pressure than in residential areas. In contrast, the A301S mutation in the *resistance to dieldrin* (*rdl*) gene, the target site of fipronil, was absent from all samples. A haplotype network analysis of all available *cytochrome c oxidase subunit I* (*COI*) sequences from GenBank, combined with those generated in this study, revealed a predominant star-like haplotype with low overall genetic diversity, likely reflecting the species’ high dispersal ability. Overall, these findings characterize the current resistance status of Turkish house flies and provide essential phenotypic and molecular data to support improved resistance management strategies.

## Introduction

The house fly, *Musca domestica* (Linnaeus, 1758) (Diptera: Muscidae), is regarded as one of the most important arthropods impacting public health and food safety, and typically constitutes nearly 90% of the fly populations in residential areas (Geden et al. 2021). House flies feed on a wide variety of substances, including milk, sugar, blood, meat broth, and numerous other foods consumed by humans (Hinkle and Hogsette, 2021). Accordingly, locations such as urban and rural areas with household waste, farmlands, livestock facilities, and slaughterhouses provide ideal environments for their survival (Larraín and Salas, 2008). Due to their frequent contact with microbe-rich animal waste and their high adult mobility, house flies are capable of spreading more than 200 pathogens affecting both humans and animals (Nayduch and Burrus, 2017; Nayduch et al. 2023).

Given the significant threat to public health posed by *Musca domestica*, extensive efforts have been made worldwide to control its populations. These efforts contribute to an estimated annual economic burden of up to $1 billion in damage and management costs (Geden et al. 2021). Despite these efforts, house flies continue to persist in high numbers within residential areas or livestock operations and pose a substantial risk to human health (Nayduch and Burrus, 2017). The primary method for controlling house flies involves the use of chemical insecticides, combined with attractants for adults, or the application of larvicides to target larval stages (Geden et al. 2021). However, control efforts often fail due to resistance development, which is driven by the repeated use of a limited number of insecticides with similar modes of action permitted in urban areas (Naqqash et al. 2016; Scott, 2017; Roca-Acevedo et al. 2023; Liu and Brown, 2026). This problem is further accelerated by the flies’ short life cycle and high fecundity, enabling populations to recover rapidly. To date, *M. domestica* has been reported to exhibit resistance to more than 66 active insecticidal compounds (Mota-Sanchez and Wise, 2025) and has been listed among the top 10 most resistant arthropod species (Sparks and Nauen, 2015).

Insecticide resistance can occur via pharmacokinetic or pharmacodynamic mechanisms in arthropods (Liu, 2015; De Rouck et al. 2023). Pharmacodynamic changes include different types of modifications at the target-site of insecticides, of which point mutations are the best-known examples (Feyereisen et al. 2015). On the other hand, pharmakinetic changes, such as alterations in ADME (Absorption, Distribution, Metabolism and Excretion), can result in a lower amount of pesticide reaching its target site (David, 2017). Besides common detoxification enzyme groups such as P450 monooxygenases, glutathione S-transferases and carboxyl/cholinesterases (Pavlidi et al. 2018; Nauen et al. 2022; Cruse et al. 2023), there is increasing attention on the novel mechanisms associated with insecticide resistance (Pu and Chung, 2024). In most cases, a combination of multiple mechanisms is involved in resistance development (Samantsidis et al. 2020).

Several chemical classes, including pyrethroids, organophosphates, carbamates, phenylpyrazoles, and neonicotinoids, have been used for house fly control (Gerry, 2025). Historically, organochlorines such as DDT and cyclodienes, including dieldrin, were used to control house flies during the 1940s–1950s (Brown, 1951). The well-known resistance mutation A301S (*D. melanogaster* numbering) in the GABA-gated chloride channel (*rdl*, resistance to dieldrin) was first identified in the house fly more than 30 years ago (Thompson et al. 1993). However, a subsequent study failed to detect this mutation in populations from the USA (Gao et al. 2007), indicating that it is not a common resistance mechanism among house fly populations worldwide.

Organophosphates (OPs) and carbamates, which target acetylcholinesterase (AChE), were widely used to control house flies between the 1960s and 1990s and are still employed in some locations (Kozaki et al. 2009). Target-site insensitivity to organophosphorus insecticides in *M. domestica* was first reported as early as the 1980s (Devonshire and Moores, 1984). In subsequent years, multiple point mutations—V260L, A316S, G342A/V, and F407Y (*Musca domestica* numbering)—in acetylcholinesterase were associated with insecticide resistance (Walsh et al. 2001; Kozaki et al. 2001).

Pyrethroid insecticides were introduced for the management of house flies in the 1980s, and resistance cases were reported soon after their introduction (Scott, 2019). The primary mechanisms of pyrethroid resistance in *Musca domestica* involve target-site mutations in the voltage-gated sodium channel (VGSC) and enhanced detoxification mediated by cytochrome P450 monooxygenases (CYPs) (Scott, 2017; Freeman and Scott, 2024). To date, multiple resistance-associated substitutions—either occurring individually or in combination—have been identified in the VGSC across diverse geographical regions worldwide, including *kdr* (L1014F), *kdr-his* (L1014H), *super-kdr* (M918T+L1014F), *Type N* (D600N+M918T+L1014F), and *1B* (T929I+L1014F) (Rinkevich et al. 2012; Kasai et al. 2017; Sun et al. 2017; Nakagawa et al. 2026).

Phenotypic and molecular resistance to insecticides in Turkish house fly populations has been investigated in several studies and decreased susceptibility to various insecticides has been consistently reported (Başkurt et al. 2011; Taşkın et al. 2011; Akıner and Çağlar, 2012; Çakır and Çetin, 2021; Cetin et al. 2023). Target-site mutations, including L1014F/H in *vgsc* and V260L, A342G, and F407Y in *ace*, have been documented in house fly populations from the Aegean and Mediterranean regions (Başkurt et al. 2011; Taşkın et al. 2011). However, the number of these studies remains limited, and continuous monitoring of both phenotypic resistance levels and the incidence of resistance mutations is needed to support the development of evidence-based resistance management programs.

In the present study, the susceptibility of house fly strains to five insecticides—permethrin, deltamethrin, bendiocarb, fipronil, and thiamethoxam—was evaluated using feeding bioassays. A comprehensive molecular screening of target-site mutations was also conducted to determine the distribution of resistance-associated alleles in house fly samples across Türkiye. Furthermore, a global genetic analysis of *cytochrome c oxidase subunit I* (*COI*) sequences from *M. domestica* was performed, and a haplotype network was constructed to assess potential phylogeographic patterns. The findings of this study provide valuable insights for the development of area-wide management strategies for house fly control.

## Materials and methods

### *Musca domestica* samples

**Fig. 1:**
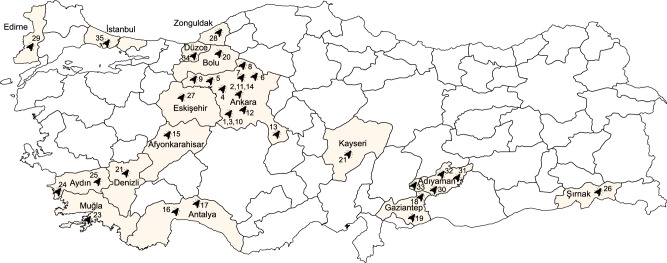
Map of study area indicating the sampling locations of 35 *Musca domestica* samples. The map was generated using mapchart.net

Five house fly strains were collected from different slaughterhouses in Ankara, Türkiye, and further propagated in climate-controlled rooms at 25 °C, 60% relative humidity, and a 16:8 (L:D) photoperiod. Adult *M. domestica* were maintained on a milk + sugar mixture, while larvae were reared in an artificial larval medium formulated per portion with 56 g wheat bran, 4 g baker’s yeast, 15 g clover pellet, 10 g milk powder, and 150 ml distilled water (modified from Cetin et al. 2009). Phenotypic resistance levels in the F1 generation of these samples were assessed as described in the bioassays section below. In addition, thirty *M. domestica* samples were collected from slaughterhouses and urban areas across the country during 2023–2024 (Fig. 1, Table S1) and subsequently preserved in 90% ethanol for molecular analysis. Including the five strains used for bioassays, a total of 35 samples were included in this study.

### Species identification and haplotype network analysis

Species identification was initially conducted through morphological examination of all samples, following the diagnostic characteristics outlined by Mullen and Durden (2009).

Genomic DNA was isolated from pooled samples, each comprising 30 adult *Musca domestica* individuals per strain, using two legs from each fly. The extraction was performed using the DNeasy Blood & Tissue Kit (Qiagen, Germany) according to the manufacturer’s protocol. In the final step, DNA was eluted in 80 µl of elution buffer. The purity and concentration of the extracted genomic DNA were assessed using a NanoDrop™ 2000 spectrophotometer (Thermo Scientific, USA). All DNA samples were stored at –20 °C until they were used for species identification and target-site mutation screening.

For molecular identification of the flies, the *cytochrome c oxidase subunit I* (*COI*) gene was amplified using the universal Folmer primers, LCO1490 and HCO2198 (see Table S2) (Folmer et al. 1994).

After the sequences were obtained, a Basic Local Alignment Search Tool (BLAST) analysis was performed in the NCBI GenBank database to confirm species identification. The *COI* sequences generated in this study, together with all available *M. domestica COI* sequences retrieved from GenBank, were used to construct haplotype networks. The sequence dataset was first trimmed, and sequences that did not overlap with the *COI* region amplified in this study were excluded in BioEdit v7.7.1 (Hall, 1999), after alignment using MAFFT v7 with default settings (Katoh et al. 2019). Genetic distances were calculated using the Kimura 2-parameter (K2P) model in MEGA 12.1 (Kumar et al. 2024). Haplotype data were generated using DnaSP v6.12.03 (Rozas et al. 2017), and the resulting haplotypes were used to construct networks in PopArt v1.7 (Leigh et al. 2015).

### Insecticides and bioassays

Feeding assays were conducted on five house fly strains following the IRAC (Insecticide Resistance Action Committee) Test Method 026 (https://irac-online.org/), with slight modifications. In brief, technical-grade permethrin (CAS:52645-53-1), deltamethrin (CAS:52918-63-5), bendiocarb (CAS:22781-23-3), fipronil (CAS:120068-37-3), and thiamethoxam (CAS:153719-23-4) were obtained from Sigma-Aldrich. All insecticides were initially dissolved in acetone; however, serial dilutions were prepared using a 20% sucrose solution for all compounds except thiamethoxam, for which acetone was used as the diluent. One milliliter (1 ml) of each insecticide solution was applied to 2 cm-long cotton dental wicks (10 mm diameter), which were then placed inside 210 ml glass vials. In case of thiamethoxam, 1 ml of insecticide solution was added to sugar cube (16 × 16 × 11 mm) and an additional moistened cotton pad was included to maintain humidity. Ten adult female flies were then introduced into each vial using CO_2_ anesthesia. All vials were incubated at 25 °C, 60% RH, 16 L:8D photoperiod. Bioassays were carried out in at least three replicates across five concentrations per insecticide. Concentrations ranging from 100 to 2500 mg/L were used for permethrin, deltamethrin, and bendiocarb. Conversely, fipronil was tested at 1.5–15 mg/L, while thiamethoxam concentrations ranged from 100 to 2000 mg/L. Acetone (for thiamethoxam) or 20% sucrose solution (for all other insecticides) was used as a control solution. Flies that could not move their legs after 48 h (72 h for thiamethoxam) were recorded as dead. Lethal concentration (LC_50_) values and their corresponding 95% confidence intervals were calculated using probit analysis via PoloPC software (LeOra Software, Berkeley, CA).

### Area-wide screening of target-site mutations in *Musca domestica* samples across Türkiye

Resistance mutations in the target genes of commonly used insecticides—*voltage-gated sodium channels* (*VGSC*), *acetylcholinesterase* (*ace*), and *GABA-gated chloride channel* (*GABACl or rdl*) were screened as described previously (Gao et al. 2007; Kozaki et al. 2009; Freeman et al. 2019). Information on the primers used for target gene amplification, along with their respective annealing temperatures, is presented in Table S2.

In addition to pooled DNA extractions from 35 fly samples (see section 2.2), individuals from populations 6, 11, and 13 were extracted separately to determine the precise allele configuration of resistance mutations. For single-fly genotyping, DNA was extracted using 30 μL of STE buffer (10 mM Tris-HCl, 100 mM NaCl, 1 mM EDTA, pH 8) and 3 μL of Proteinase K (10 mg/mL; Sigma-Aldrich). Each fly was homogenized in the buffer mixture and incubated at 60 °C for 30 min, followed by Proteinase K inactivation at 95 °C for 5 min. The resulting crude DNA was used as a template for subsequent PCR reactions.

Each PCR reaction was carried out in a final volume of 30 μl, which included 2 μl of fly DNA (with a concentration between 40 and 80 ng/μL), 1 μl each of forward and reverse primers, 11 μl of nuclease-free ultrapure water, and 15 μl of EmeraldAmp® MAX Master Mix (Takara Bio Inc., Japan). Post-PCR products were verified by agarose gel electrophoresis and subsequently sequenced at BM Company (Ankara, Türkiye).

Sequencing chromatograms were visually inspected to identify resistance-associated mutations using BioEdit v7.7.1 (Hall, 1999).

### Molecular docking of wild-type and mutant *Musca domestica* acetylcholinesterase

The three-dimensional structure of wild-type and mutant *Musca domestica* acetylcholinesterase (AChE) was predicted using AlphaFold3 based on the amino acid sequence (accession number: AF281161) to generate a high-confidence model of the wild-type protein (Jumper et al. 2021; Abramson et al. 2024). Mutant AChE structures were generated by introducing specific amino acid substitutions into the validated wild-type model; mutants were named according to the substituted residues and positions (e.g., VGF for V260, G342, F407). The predicted structure was validated using the SAVES v6.1 web interface (https://saves.mbi.ucla.edu/), including ERRAT and PROCHECK analyses, to assess stereochemical quality and overall structural reliability before docking. Molecular docking was performed using CB-Dock (Liu et al. 2020), an automated docking server that identifies potential binding cavities and performs docking through the AutoDock Vina algorithm. The ligands, acetylcholine and bendiocarb, were submitted as SMILES strings retrieved from PubChem, and binding energies (kcal/mol) were calculated for each predicted protein–ligand complex. The top-ranked docking poses with the lowest binding energies were selected for further analysis. Protein–ligand interactions were visualized using PyMOL (DeLano, 2002) to illustrate the binding orientations and interaction sites within the active pocket.

## Results

### Species identification and haplotype network analysis

**Fig. 2:**
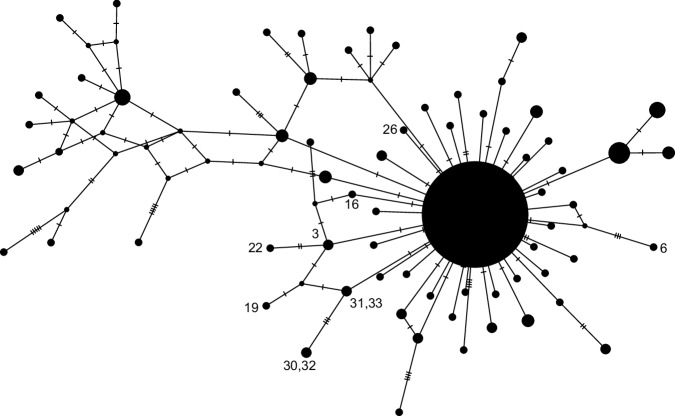
Haplotype network analysis based on COI sequences of worldwide samples of *Musca domestica*, with circle sizes proportional to haplotype frequency. Samples that do not belong to the predominant haplotype are shown

All field-collected samples were identified as *Musca domestica* based on morphological characteristics. Molecular analysis further confirmed the morphological identification of all samples as *M. domestica*. The obtained COI sequences (accession numbers: PZ299031-PZ299065) showed over 98% similarity to previously published *M. domestica* COI sequences. The mean genetic distance among global *M. domestica* COI sequences was calculated as 0.23%, with 558 nucleotides conserved across global populations. In comparison, slighty higher genetic variation (0.28%) was observed in Turkish samples, based on sequences generated in this study and those retrieved from GenBank (OM541932–OM541940). Sequence analysis of 35 samples from Türkiye revealed nine distinct haplotypes, with one predominant haplotype occurring in 25 Turkish samples as well as in most other samples (Fig. 2).

### Bioassays

The results of the toxicity bioassays are summarized in Table 1. Pyrethroids (permethrin and deltamethrin) were ineffective against all *M. domestica* strains, with LC_50_ values exceeding 2500 mg a.i./L. Three strains displayed LC_50_ values for bendiocarb exceeding 2500 mg a.i./L, whereas the Yenimahalle and Sincan samples had LC_50_ values of 1541.86 and 1321.04 mg a.i./L, respectively. The efficacy of thiamethoxam varied considerably among five strains and the LC_50_ values ranging from 116. mg a.i./L (Polatlı) to 620.30 mg a.i./L (Yenimahalle), reflecting heterogeneous susceptibility levels. The most effective insecticide was fipronil, with LC_50_ values between 2.81 mg a.i./L (Sincan) and 7.13 mg a.i./L (Yenimahalle).

**Table 1:** LC_50_ values of five insecticides for field-collected *Musca domestica* strains in Türkiye

Insecticide	Strain	Slope+SE	LC_50_ (mg a.i./L) (95% CL)	df	χ^2^	RR^a^
Permethrin	Kazan (7)^b^	>2500				-
	Yenimahalle (12)	>2500				-
	Çubuk (6)	>2500				-
	Sincan (11)	>2500				-
	Polatlı (10)	>2500				-
Deltamethrin	Kazan	>2500				-
	Yenimahalle	>2500				-
	Çubuk	>2500				-
	Sincan	>2500				-
	Polatlı	>2500				-
Fipronil	Kazan	4.066 ± 0.907	5.14 (3.79–6.21)	13	12.2	1.8
	Yenimahalle	3.141 ± 0.617	7.13 (5.49–8.77)	13	7.9	2.5
	Çubuk	3.329 ± 0.668	6.85 (4.56–8.95)	13	12.2	2.4
	Sincan	1.270 ± 0.353	2.81 (1.11–4.37)	13	11.8	-
	Polatlı	2.746 ± 0.508	4.61 (3.35–5.85)	13	4.4	1.6
Bendiocarb	Kazan		>2500			>1.9
	Yenimahalle	7.054 ± 2.341	1541.86 (1087.90–1828.92)	13	4.9	1.1
	Çubuk	>2500				>1.9
	Sincan	4.349 ± 1.097	1321.04 (1029.87–1566.25)	13	3.5	-
	Polatlı	>2500				>1.9
Thiamethoxam	Kazan	1.637 ± 0.397	384.97 (201.10–551.12)	13	2.0	2.4
	Yenimahalle	2.233 ± 0.418	620.30 (455.44–826.37)	13	7.8	3.8
	Çubuk	1.709 ± 0.401	392.28 (210.60–560.61)	13	2.9	2.4
	Sincan	2.406 ± 0.390	594.62 (455.50–767.45)	13	3.7	3.7
	Polatlı	1.205 ± 0.271	161.52 (65.83–259.10)	13	7.8	-

^a^RR: Resistance ratio: LC_50_ of certain strain / LC_50_ of most susceptible strain.

^b^Numbers correspond to those listed in Table S1.

### Screening of resistance mutations

An overview of the detected target-site mutations is presented in Table 2. All samples were screened for mutations in the *vgsc*, *rdl*, and *ace* genes, which are the respective targets of pyrethroids, fipronil, and organophosphates/carbamates. The *kdr* (L1014F) mutation was detected in 29 samples, however, the mutation was fixed in only one sample. The *kdr-his* mutation was found in five samples, all of which were collected from slaughterhouses where deltamethrin exposure was common (see Table S2). The T929I mutation was identified in 17 samples but was not fixed in any of them. No mutations were detected at positions 918 or 600 in the *vgsc* gene. The A301S mutation in the *rdl* gene was not detected in any of the fly samples.

**Table 2:** Amino-acid substitutions at the resistance-associated positions in *vgsc, rdl* and *ace* of 35 *Musca domestica* samples. Multiple alleles are listed in order of decreasing estimated frequency

Pop	VGSC				RDL	AChE			
	M918	T929	D600	L1014	A301	V260	A316	G342	F407
1	M	T/I	D	F/L	A	L/V	A	A/G	Y
2	M	T/I	D	F/L	A	V	A	G	Y
3	M	T/I	D	F/L	A	V/L	A	G/A	Y
4	M	T/I	D	L/F/H	A	V/L	A	G/A	Y
5	M	T/I	D	L/F	A	V/L	A	G/A/V	Y
6^a^	M	T	D	L/H/F	A	V/L	A	A/G/V	Y
7^a^	M	T/I	D	L/F	A	V/L	A	G/A/V	Y
8	M	T/I	D	F/L/H	A	L/V	A	A/G	Y
9	M	T/I	D	L/F	A	V	A	G/A/V	Y
10^a^	M	T/I	D	L/F	A	V	A	G/A	Y
11^a^	M	T/I	D	F/L/H	A	V/L	A/S	A/G	Y
12^a^	M	T/I	D	L/F	A	V/L	A	G/A/V	Y
13	M	T/I	D	F/H/L	A	V/L	A	A/G	Y
14	M	T	D	F/L	A	V	A	G/A	Y
15	M	T/I	D	F/L	A	V	A/S	G/A	Y
16	M	T	D	F/L	A	V/L	A	A/G	Y
17	M	T	D	F/L	A	V/L	A	A/G	Y
18	M	T	D	F/L	A	V/L	A	G	Y
19	M	T	D	L	A	V/L	A	G/A	F
20	M	T/I	D	F/L	A	V	A	G	Y
21	M	T	D	F/L	A	V/L	A	G/A	Y
22	M	T	D	F/L	A	V	A	G	Y
23	M	I/T	D	F/L	A	V/L	A	G/A	Y
24	M	T/I	D	F/L	A	V/L	A	G/A	Y
25	M	T	D	L	A	V	A/S	G	Y
26	M	T	D	F	A	V	A	G	Y
27	M	T/I	D	F/L	A	V/L	A	G/A	F
28	M	T	D	F/L	A	V/L	A	G	Y
29	M	T	D	L	A	V	A	G	Y/F
30	M	T	D	L	A	V/L	A	A	Y
31	M	T	D	L	A	V/L	A	A	Y
32	M	T	D	F/L	A	V/L	A	A	Y
33	M	T	D	F/L	A	V/L	A	A	Y
34	M	T	D	F/L	A	V/L	A	G/A	Y
35	M	T	D	L	A	V/L	A	G/A	F/Y

^a^Strains used in bioassays.

The V260L and A316S mutations in *ace* were found in 25 and 3 samples, respectively, but neither mutation was fixed in any of them. The G342A mutation was present in 27 samples, and it was fixed in four of them. The G342V mutation was detected in only five samples, all of which also carried the G342A mutation. Notably, all G342V samples originated from slaughterhouses. Last, the F407Y mutation was the most frequent, detected in 33 out of 35 samples, and was fixed in all samples except two.

The genotypes of individual flies from populations 6, 11, and 13 are summarized in Table S3. Most individuals were homozygous for the F407Y mutation, whereas over half were heterozygous at positions L1014, V260, and G342. Notably, the 1014H (histidine) allele was always found in a heterozygous state, occurring in combination with either the 1014F (phenylalanine) or the 1014L (leucine) allele in all examined individuals.

### Molecular docking of wild-type and mutant *Musca domestica* acetylcholinesterase

The wild-type AChE structure was validated using Ramachandran plot and ERRAT analysis (86.5% residues in favored regions, ERRAT score 90.2) as a representative example. All mutant models were generated based on this validated structure, ensuring reliability for subsequent docking analyses. The binding residues for both acetylcholine and bendiocarb are presented in Table S4.

**Fig. 3:**
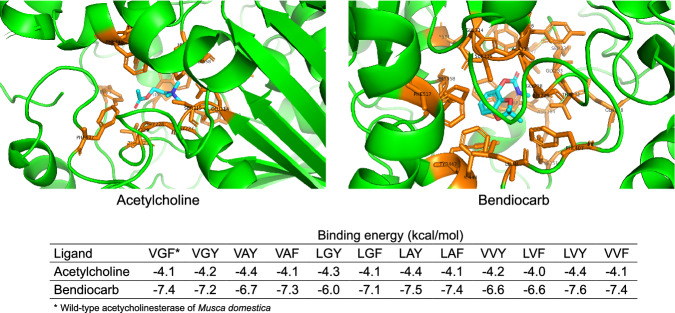
Molecular docking of acetylcholine and bendicarb with *Musca domestica* AChE, showing wild-type (VGF: V260, G342, F407) binding residues and predicted binding energies for both wild-type and mutant enzymes

Acetylcholine binding was evaluated using the crystal structure of the mutant S203A mouse acetylcholinesterase complexed with acetylcholine (PDB: 2HA4), which was automatically selected as the best template by CB-Dock2. The model indicated that Ser315 and His557, which constitute part of the catalytic triad, together with the known resistance mutation sites such as Phe407 and Phe448 (Cruse et al. 2023), are located within the binding site (Table S4), thereby further validating the predicted structure. The predicted binding energy of acetylcholine to the wild-type acetylcholinesterase was −4.1 kcal/mol. Overall, the binding scores of the mutant AChEs were similar to those of the wild type, although slightly stronger binding was observed for variants such as VAY, LAY, and LVY (Fig. 3).

For bendiocarb, the mutant variants generally exhibited lower binding scores than the wild type, suggesting reduced affinity of the mutant AChEs for bendiocarb (Fig. 3). A few variants, such as LAY and LVY, showed scores close to the wild type. Notably, the LGY, VVY, LVF, and VAY variants displayed the most pronounced reductions in bendiocarb binding (Fig. 3).

## Discussion

Genetic analyses using *M. domestica* COI sequences from populations across the globe revealed very low genetic distances between populations. Although slightly higher variation was observed in Turkish flies, overall diversity remained low, consistent with Doğaç (2016). While several partial studies have been conducted (Pastor et al. 2014; Doğaç, 2016), to the best of our knowledge, no comprehensive haplotype network analysis has been performed so far. The observed low variation is further illustrated by the haplotype network analysis, which shows a dominant central haplotype surrounded by a few low-frequency variants. Such a pattern likely reflects the species’ high dispersal capacity, enabling individuals to overcome geographic barriers and maintain extensive gene flow, thereby preventing substantial genetic divergence despite its long evolutionary history. This high dispersal ability may also contribute to the global similarity of insecticide resistance mechanisms observed in *M. domestica* populations.

House fly control is often compromised by various factors with insecticide resistance being the primary cause (Scott, 2017; Geden et al. 2021). Various levels of resistance have been documented in Turkish strains, highlighting the necessity of continuous monitoring of resistance and major resistance mechanisms to design effective resistance management programs.

Pyrethroids are widely used in urban pest control, including against house flies, due to their low environmental persistence and relatively low mammalian toxicity (Maund et al. 2011; Tsuji et al. 2011). Permethrin was the first pyrethroid applied against house flies, followed by the registration of many others (Roca-Acevedo et al. 2023). Resistance to pyrethroids has been mainly driven by mutations in the voltage-gated sodium channel (VGSC), the target site of these compounds, across arthropods (Rinkevich et al. 2012; Scott, 2019), although other mechanisms, such as increased detoxification enzyme activity, have also been reported (Scott, 2017; Ye et al. 2022; You et al. 2023; Freeman and Scott, 2024).

Although residual contact assays have been commonly used in previous studies, the present study employed a feeding method to assess insecticide efficacy and evaluate their potential in attract-and-kill–based control. This bait-based approach is particularly critical in locations where direct spraying is not feasible, such as slaughterhouses. Several studies have reported that pyrethroids, including deltamethrin, can be effective against dipteran species, including houseflies, when administered via feeding (Khan et al. 2013; Khan and Akram, 2014; Snyder et al. 2016). In contrast, the present results showed that pyrethroids (deltamethrin and permethrin) were ineffective in the feeding assay, with LC_50_ values exceeding 2500 mg/L. This inefficacy is likely attributable to the inherently low activity of pyrethroids when ingested, given their primary mode of action as contact insecticides (Katsuda, 2011). Although resistance mutations are present in the tested strains, the observed lack of efficacy is more consistent with limited feeding activity rather than resistance alone.

In *Musca domestica*, pyrethroid resistance is likely rooted in the historical use of DDT, a VGSC-targeting organochlorine insecticide, which selected for the L1014F kdr mutation (Scott, 2017). Afterwards, the sequential evolution of combinations of several mutations, including M918T+L1014F and D600N+M918T+L1014F, has been associated with resistance (Scott, 2019). Additionally, a histidine variant of L1014F (L1014H, *kdr-his*) has been reported, though it confers only low levels of permethrin resistance (Liu and Pridgeon, 2002). The T929I mutation, in combination with L1014F, has been documented in house flies from the USA (Kasai et al. 2017) and functional studies using electrophysiology and CRISPR-Cas9 in other dipterans have validated the roles of both T929I and L1014F in pyrethroid resistance (Vais et al. 2001; Usherwood et al. 2007; Grigoraki et al. 2021).

In the present study, none of the screened samples carried the M918T or D600N mutations, consistent with Taşkın et al. (2011), who reported the absence of the *super-kdr* mutation in Turkish house fly samples. The *kdr* mutation, however, was highly prevalent across the samples; although it was fixed in only one sample, and only six out of 35 samples remained fixed for the wild-type 1014L allele. Compared to samples collected ~20 years ago (Taşkın et al. 2011), those in the present study show a marked increase in *kdr* frequency, reflecting intensified pyrethroid use. The absence of fixation of *kdr* mutations may be explained by fitness costs associated with these mutations; however, such costs can vary among different *kdr* haplotypes (Rinkevich et al. 2013). Therefore, future studies are needed to investigate the fitness effects of *kdr* variants in Turkish house fly strains. Five strains collected from slaughterhouses, which experienced higher selection pressure than urban areas with less treatment, carried *kdr-his* together with *kdr* and the susceptible leucine allele. This pattern may result from intense selection pressure driving the evolution of stronger resistance-conferring mutations. Although it has been suggested that the phenotypic strength of VGSC mutations follows *kdr-his*<*kdr*<*super-kdr* (Scott, 2017), electrophysiological studies in *Xenopus* oocytes indicate that L1014H can have a stronger effect than L1014S/F against deltamethrin (Burton et al. 2011), which was widely used in the sampled areas. Furthermore, *wild-type*/*kdr-his* and *kdr*/*kdr-his* haplotypes exhibit incomplete dominance against deltamethrin (Sun et al. 2016), which could facilitate the evolution of the *kdr-his* haplotype. Moreover, seventeen of the *kdr*-carrying samples also harboured the T929I mutation. As pooled DNA samples were used for genotyping, the specific allelic configurations and mutation combinations could not be precisely resolved for the majority of the samples. However, individual genotyping of three selected samples (6, 11, and 13) revealed that neither the T929I nor the kdr-his mutation was found in a homozygous state in any individual. Although the mutations were not fixed in most samples, repeated selection could lead to fixation over a short time, potentially further compromising pyrethroid efficacy. On the other hand, the absence of fixation is encouraging, as crossing with wild-type alleles will dilute resistant individuals. Given the partially recessive nature of pyrethroid resistance mutations, heterozygotes display a susceptible phenotype (Sun et al. 2016). Therefore, pyrethroids could still be included in rotation programs, albeit with caution. Recent empirical data suggest that a 5% mutation frequency represents the threshold for the emergence of pyrethroid resistance in spider mites (Yang et al. 2024). A similar approach could be applied to house flies to enable molecular diagnostics for resistance in future studies.

Bendiocarb is a carbamate insecticide that targets acetylcholinesterase and is frequently used against various urban pests including house flies and mosquitoes since the early 1970s (Lemon and Bromilow, 1977). Although its long-term use, the knowledge on its feeding activity is very limited (Luong et al. 2022). The toxicity of bendiocarb was low on tested strains. However, further studies are needed to uncover if this observed decreased susceptibility is due to low feeding activity of bendiocarb or development of resistance as a result of long-term use of OPs.

Similar to pyrethroids, point mutations at the target site of bendiocarb, AChE, were suggested to be the primary reason for resistance development in house flies. Mutations at position 342 and 407 were previously identified in different dipteran species, *Drosophila* and *Aedes* as well as *M. domestica* (Fournier, 2005). In addition, *ace* duplication was only recently documented in *M. domestica* populations (Nakagawa et al. 2026). This may have emerged as a potential mechanism to offset the fitness costs associated with resistance, a phenomenon also observed in other arthropod species (Lee et al. 2015). Although the role of target-site mutations in conferring AChE insensitivity to several OPs such as fenitroxon, paraoxon and malaoxon was demonstrated through biochemical assays in *M. domestica* (Chen et al. 2001; Kozaki et al. 2001), the effect of these mutations may vary between AChE inhibitors (Fournier, 2005). For bendiocarb, G262A alone, when expressed in a baculovirus system, resulted in a 3.1-fold insensitivity, however, its presence in combination with F407Y further increased the effect to 5.2-fold (Walsh et al. 2001). On the other hand, the G262V mutation alone, can result in far more striking inhibition (85-fold) than its G262A variant and thus could be a major mechanism (Walsh et al. 2001). In this study, in silico modelling using *M. domestica* AChE showed that the VAY, LGY, VVY, and LVF variants exhibited significantly lower predicted binding scores than the wild-type VGF, underscoring the importance of mutation combinations rather than individual substitutions. Contrary to the empirical findings of Walsh et al. (2001), the G262V mutation alone did not reduce the predicted binding affinity in our in silico analysis. This discrepancy highlights the importance of caution when interpreting computational predictions without direct empirical validation. While computational modeling provides valuable insights, our results demonstrate that these predictions must be confirmed with experimental data. Nevertheless, two variants containing this mutation (VVY and LVF) displayed substantially reduced binding, suggesting that G262V might have a stronger impact on resistance when combined with other mutations.

In the present study, the F407Y mutation was fixed in more than 85% of all samples, and 24 out of 33 harbored G342A/V, indicating a high prevalence of insensitive AChE in Turkish house fly samples. Individual genotyping from three representative populations further supported these results. However, although certain mutations (e.g., A301S and F407Y) appeared to be either fixed or absent based on pooled analysis, individual genotyping revealed their presence at low frequencies in a few samples. This discrepancy highlights the critical importance of individual genotyping, as Sanger sequencing of pooled DNA often fails to detect rare alleles when their frequency falls below the 10–20% threshold (Njiru et al. 2023; İnak et al. 2024). In line with this, a very high frequency of the F407Y mutation has been documented across various countries (Adib et al. 2023; Alzabib et al. 2023; Yang et al. 2023; You et al. 2025). Although F407Y alone causes only partial insensitivity, its effect increases when combined with other mutations (Walsh et al. 2001), as observed in our study, where G342V was consistently found alongside F407Y. These results align with Başkurt et al. (2011), who reported a high prevalence of F407Y in Turkish house flies and noted that G342V was always present with F407Y. In addition, the A316S mutation, initially reported by Kozaki et al. (2009), was detected in three out of 35 samples and was also found in a few individuals from populations 6 and 11. To the best of our knowledge, this represents the first report of this mutation in Turkish house fly populations. Similar to *kdr-his*, the G342V mutation, which confers the highest insensitivity to bendiocarb (Walsh et al. 2001), was found only in slaughterhouse samples, likely due to higher selection pressure in these areas. Likewise, Alzabib et al. (2023) reported the G342V mutation in *M. domestica* populations from Saudi Arabia, albeit at a low frequency. However, because that study did not include samples from outside slaughterhouses, it remains unclear whether the G342V mutation is uniquely restricted to those environments, as observed in our findings. The F407Y mutation has also been shown to compensate for the fitness cost associated with G342A/V mutations (Walsh et al. 2001). Therefore, the fixation of F407Y in most samples indicates that susceptibility is unlikely to be restored. Overall, these findings suggest that the use of bendiocarb or other AChE inhibitors for house fly control is inadvisable.

Fipronil, belonging to phenylpyrazole group, is another insecticide that is used in control of house flies as well as several agricultural pests (Kristensen et al. 2004). Several studies showed the high efficacy of fipronil against *M. domestica* (Scott and Wen, 1997; Scott et al. 2000; Kristensen et al. 2004). However, the risk for resistance development was also documented (Abbas et al. 2014). In the present study, fipronil exhibited the highest efficacy in feeding assays, indicating its strong potential for house fly control via attract-kill approach. In Türkiye, the use of fipronil—except for gel formulations—was discontinued in 2020, and its importation and manufacturing were terminated at the beginning of 2024 (Anonymous, 2020; 2023). This high efficacy could therefore be associated with a dominant fitness cost of fipronil resistance in *M. domestica*, resulting in a significant decrease in resistance after five generations (Abbas et al. 2016). These findings not only highlight the impact of selection pressure removal on resistance dynamics but also provide valuable insights for designing integrated pest management programs, emphasizing the rotation of insecticides with different modes of action to restore susceptibility and delay resistance development in house fly populations.

An alanine-to-serine substitution at position 301 in the second membrane-spanning domain (*Drosophila melanogaster* numbering) was first characterized in association with cyclodiene resistance, and the corresponding gene was named *resistance to dieldrin* (*Rdl*), a GABA-gated chloride channel (ffrench-Constant et al. 2000). The same A301S mutation also conferred a low to moderate resistance to fipronil, aiming for the same target gene (Remnant et al. 2014; Guest et al. 2019). Although this mutation and its variants have been frequently uncovered in a wide range of arthropods (Feyereisen et al. 2015), it has been rarely found in *M. domestica* (Thompson et al. 1993). Indeed, Gao et al. (2007) reported the absence of this mutation in house fly populations from the USA. Consistent with their findings and with our bioassay results, we also detected no A301S mutation in any of the samples. One possible explanation for its absence in house flies is a potential fitness cost associated with the A301S substitution. However, no evidence of such a cost has been reported for this mutation in this species. In contrast, the mutation persists at high frequencies in several other insect species, even in the absence of insecticide pressure, suggesting that it imposes little or no fitness cost (Thompson et al. 1993). The absence of the A301S mutation in our samples suggests that target-site resistance via the RDL receptor is not a prevalent mechanism in Turkish house fly samples. If resistance were to emerge, it would more likely arise through metabolic mechanisms involving the overexpression of detoxification enzymes; consistent with this, studies in *M. domestica* have shown that synergism by PBO and DEF— inhibitors of P450s and CCEs, respectively—enhances the toxicity of fipronil (Liu and Yue, 2000). These findings highlight the need to monitor metabolic resistance pathways in house fly populations, as they may play a more prominent role than target-site mutations in mediating fipronil resistance.

Thiamethoxam is one of the most widely used insecticides, belonging to the neonicotinoid class, and is commonly applied for house fly control in bait formulations that include the housefly pheromone cis-tricos-9-en. This group of insecticides acts as competitive modulators of nicotinic acetylcholine receptors (Taillebois et al. 2018). Decreased susceptibility to thiamethoxam has been documented worldwide. Similarly, Turkish house flies have shown high levels of resistance to thiamethoxam (Çakır and Çetin, 2021; Cetin et al. 2023). In the present study, variable susceptibility was also observed, in line with the widespread use of thiamethoxam in the country. Thiamethoxam in bait formulations is a good option for locations where direct spraying is not feasible, such as restaurants and slaughterhouses. Therefore, relying solely on this compound with repeated use should be avoided.

In conclusion, the present study demonstrates the widespread occurrence of target-site mutations and the presence of house fly strains exhibiting multiple resistance phenotypes. Notably, the high resistance levels observed in slaughterhouse collections highlight these facilities as critical hotspots for resistance development due to intensive insecticide selection pressure. To maintain insecticide efficacy and mitigate public health risks, alternative control strategies and integrated resistance management programs must be prioritized.

## Supplementary information

Supplementary Tables

## Data Availability

All data supporting the findings of this study are available within the paper and its Supplementary Information.
