# Supplementary material for: Insecticide resistance level and geographical distribution of target-site mutations in field collections of the house fly (Musca domestica) from Türkiye
Source: Ecotoxicology. 2026 Jun 18;35(5):123. doi: 10.1007/s10646-026-03107-7 (PMC13279603; doi:10.1007/s10646-026-03107-7)
Supplement: Supplementary file 1 — Supplementary Tables [file 10646_2026_3107_MOESM1_ESM.docx]

Table S1. Detailed information on field- collected *Musca domestica* samples

| **No** | **Location** | **S/U^a^** | **Sampling date** | **Last insecticide applications** |
| --- | --- | --- | --- | --- |
| 1 | Ankara/Polatlı | S | Sept 2023 | Deltamethrin, tetramethrin, cypermethrin, |
| 2 | Ankara/Sincan | S | Sept 2023 | Thiamethoxam |
| 3 | Ankara/Polatlı | S | Sept 2023 | Thiamethoxam |
| 4 | Ankara/Ayaş | S | Sept 2023 | Permethrin, deltamethrin |
| 5 | Ankara/Beypazarı | S | Sept 2023 | Thiamethoxam, Deltamethrin |
| 6* | Ankara/Çubuk | S | Sept 2023 | Deltamethrin |
| 7* | Ankara/Kazan | S | Sept 2023 | Thiamethoxam |
| 8 | Ankara/Kızılcahamam | S | Sept 2023 | Deltamethrin, Thiamethoxam |
| 9 | Ankara/Nallıhan | S | Sept 2023 | Deltamethrin, permethrin, tetramethrin |
| 10* | Ankara/Polatlı | S | Oct 2024 | Alpha-cypermethrin |
| 11* | Ankara/Sincan | S | Sept 2023 | Permethrin, deltamethrin tetramethrin |
| 12* | Ankara/Yenimahalle | S | Sept 2023 | Thiamethoxam |
| 13 | Ankara/Şereflikoçhisar | S | Sept 2023 | Deltamethrin, Thiamethoxam |
| 14 | Ankara/Sincan | S | Aug 2024 | Permethrin |
| 15 | Afyon/Centrum | U | Aug 2024 | Unknown |
| 16 | Antalya/Döşemealtı | U | June 2024 | Unknown |
| 17 | Antalya/Centrum | U | June 2024 | Unknown |
| 18 | Gaziantep/Araban | U | Sept 2024 | Unknown |
| 19 | Gaziantep/Centrum | U | July 2024 | Unknown |
| 20 | Bolu/Centrum | U | Sept 2024 | Unknown |
| 21 | Denizli/Centrum | U | Aug 2024 | Unknown |
| 22 | Kayseri/Centrum | U | Sept 2024 | Unknown |
| 23 | Muğla/Selimiye | U | July 2024 | Unknown |
| 24 | Aydın/Didim | U | July 2024 | Unknown |
| 25 | Aydın/Centrum | U | July 2024 | Unknown |
| 26 | Şırnak/Centrum | U | Aug 2024 | Unknown |
| 27 | Eskişehir/Centrum | U | June 2024 | Unknown |
| 28 | Zonguldak/Centrum | U | June 2024 | Unknown |
| 29 | Edirne/Centrum | U | Aug 2024 | Unknown |
| 30 | Adıyaman/Centrum | U | Sept 2024 | Unknown |
| 31 | Adıyaman/Besni | U | Sept 2024 | Unknown |
| 32 | Adıyaman/Kahta | U | Sept 2024 | Unknown |
| 33 | Adıyaman/Şambayat | U | Sept 2024 | Unknown |
| 34 | Düzce/Centrum | U | Oct 2024 | Unknown |
| 35 | İstanbul/Büyükçekmece | U | Sept 2024 | Unknown |
| ^a^ Slaughterhouse/Urban areas | | | | |
| ^*^ Strains used in bioassays | | | | |

Table S2. Sequences, amplicon sizes, and annealing temperatures of primers used in this study

| **Gene** | **Genotype** | **Primer** | **Sequence (**5′- 3′) | **Amplicon size (bp)** | **Annealing temperature (^o^C)** | **Reference** |
| --- | --- | --- | --- | --- | --- | --- |
| *vgsc* | L1014H/F | kdrFL  MdSCR7 | TCGCTTCAAGGACCATGAATTACCGCGCTG  TGGTATCATTGTCGGCAGTC | 260 | 57 | Freeman et al., 2019 |
|  | M918T, T929I | MdSCF52  MdSCR3 | GCAAAATCATGGCCCACACT  GTTCTTTCCGAAAAGTTGCATTCC | 138 | 57 | Freeman et al., 2019 |
|  | D600N | MdSCF61  MdSCR62 | AATACGAAATGGGCGTGGAC  CATTCTCTTCGGACATTGGTG | 140 | 57 | Freeman et al., 2019 |
| *ace* | V260L, A316S, G342A/V, F470Y | S90MdAce  AS89MdAce | CATCTAAAACCGATCAGGACCATTTAATAC  TCATCTTTAACATTTCCAATCAGAATATCG | 824 | 55 | Kozaki et al., 2009 |
| *rdl* | A301S | Md_Rdl_F2  Md_Rdl_R2 | TCTTACAGGAAATTATTCGCGTC  ACTGGCAAAGACCATCACGAAACAC | 289 | 55 | Gao et al., 2007 |
| *COI* |  | LCO1490  HCO2198 | GGTCAACAAATCATAAAGATATTGG  TAAACTTCAGGGTGACCAAAAAATCA | 680 | 48 | Folmer et al., 1994 |

Table S3. Genotyping of indivudial flies from sample 6, 11, 13 for resistance mutations.

| **Strains** | **VGSC** | | | | **RDL** | **AChE** | | | |
| --- | --- | --- | --- | --- | --- | --- | --- | --- | --- |
|  | **M918** | **T929** | **D600** | **L1014** | **A301** | **V260** | **A316** | **G342** | **F407** |
| 6-1 | M | T | D | L | A | V/L | A | A | Y |
| 6-2 | M | T | D | L/H | A | V/L | A | G/V | Y |
| 6-3 | M | T | D | L | A | V/L | A | A | Y |
| 6-4 | M | T | D | L | A | V/L | A | A | Y |
| 6-5 | M | T | D | F | A | V/L | A | A | Y |
| 6-6 | M | T/I | D | F/H | A | V/L | A/S | G/V | Y |
| 6-7 | M | T | D | L/H | A | V/L | A | G/A | Y |
| 6-8 | M | T | D | L | A | V/L | A | A | Y |
| 6-9 | M | T | D | L | A | V/L | A | A | Y |
| 6-10 | M | T | D | L | A | V/L | A | A | Y |
| 11-1 | M | T | D | F/H | A | V/L | A | A | F/Y |
| 11-2 | M | T/I | D | F/H | A | V | A | G/A | Y |
| 11-3 | M | T/I | D | L | A | V | A | G | Y |
| 11-4 | M | T/I | D | L/F | A | V/L | A | G/A | Y |
| 11-5 | M | T/I | D | L/F | A | V/L | A | G/A | Y |
| 11-6 | M | T | D | L/H | A | V/L | A/S | G/A | F/Y |
| 11-7 | M | T/I | D | F/H | A | V/L | A | G/A | Y |
| 11-8 | M | T | D | L | A | V | A/S | G/A | Y |
| 11-9 | M | T | D | L | A | V/L | A | A | Y |
| 11-10 | M | T/I | D | L/F | A | V/L | A/S | G/A | Y |
| 13-1 | M | T/I | D | F/H | A | V/L | A | A | Y |
| 13-2 | M | T | D | L | A | V | A | G/A | Y |
| 13-3 | M | T/I | D | F/H | A | V | A | G | Y |
| 13-4 | M | T | D | F/H | A | V/L | A | G/A | Y |
| 13-5 | M | T/I | D | L/F | A | V/L | A | G/A | Y |
| 13-6 | M | T/I | D | L/H | A | V/L | A | G/A | Y |
| 13-7 | M | T/I | D | F/H | A | V/L | A | G/A | Y |
| 13-8 | M | T | D | F/H | A | V | A | G/A | Y |
| 13-9 | M | T | D | F/H | A | V/L | A | A | Y |
| 13-10 | M | T/I | D | L/F | A | V/L | A | G/A | F/Y |

Table S4. Binding residues of acetylcholine and bendicarb in the wild-type acetylcholinesterase of *Musca domestica*.

| Acetylcholine | Bendiocarb |
| --- | --- |
| GLN148 TYR151 GLY159 ILE162 TRP163 GLY227 GLY228 GLY229 THR232 GLY233 LEU237 ILE239 TYR240 GLU314 SER315 TRP348 LEU405 PHE407 TYR447 PHE448 PHE517 THR544 THR546 SER547 LEU548 TRP549 LEU556 HIS557 GLY558 ASP559 ILE561 GLU562 | GLN148 GLU149 ARG150 TYR151 SER158 GLY159 ILE162 TRP163 ASN164 PRO165 TYR226 GLY227 GLY228 GLY229 THR232 GLY233 SER234 LEU237 ILE239 TYR240 GLU314 SER315 ALA316 TRP348 TRP398 TYR401 LEU405 PHE407 TYR447 PHE448 TYR451 PHE517 THR544 THR546 SER547 LEU548 TRP549 LEU556 HIS557 GLY558 ASP559 ILE561 GLU562 GLN567 TYR575 |
